# Application of a natural language processing algorithm to early asthma ascertainment for adults in the era of electronic health records

**DOI:** 10.1016/j.jacig.2025.100618

**Published:** 2025-11-26

**Authors:** Chung-Il Wi, Thanai Pongdee, Hee Yun Seol, Sunghwan Sohn, Elham Sagheb, Bhavani Singh Agnikula Kshatriya, Shauna M. Overgaard, Deepak K. Sharma, Sungrim Moon, Elizabeth A. Krusemark, Dave Watson, Sergio E. Chiarella, Miguel A. Park, Jason D. Greenwood, Randy M. Foss, Zhandong Liu, Meera Gupta, Carla M. Davis, Wade Schulz, Hongfang Liu, Young J. Juhn

**Affiliations:** a Department of Pediatric and Adolescent Medicine, Mayo Clinic, Rochester, Minn; b Precision Population Science Lab, Mayo Clinic, Rochester, Minn; c Division of Allergic diseases, Mayo Clinic, Rochester, Minn; d Department of Artificial Intelligence and Informatics, Mayo Clinic, Rochester, Minn; e Center for Digital Health, Mayo Clinic, Rochester, Minn; f Division of Clinical Trial and Biostatistics, Mayo Clinic, Rochester, Minn; g Department of Family Medicine, Mayo Clinic, Rochester, Minn; h Department of Internal Medicine, Mayo Clinic, Rochester, Minn; i Department of Internal Medicine, Pusan National University Yangsan Hospital, Yangsan, Korea; j Family Medicine, Mayo Clinic Health System, Lake City, Minn; k Department of Computational Sciences, Texas Children’s Hospital, Houston, Tex; l Division of Immunology, Allergy, and Retrovirology, Baylor College of Medicine, Houston, Tex; m Department of Pediatrics and Child Health, Howard University, Washington, DC; n Department of Informatics, Laboratory Medicine, Yale School of Medicine, New Haven, Conn; o McWilliams School of Biomedical Informatics, University of Texas Health Science Center at Houston, Houston, Tex

**Keywords:** Asthma, adult, natural language processing, diagnosis, electronic health record, diagnosis management, artificial intelligence, algorithm

## Abstract

**Background:**

The natural language processing (NLP) algorithm for predetermined asthma criteria (NLP-PAC) was successfully developed and validated for automatically ascertaining pediatric asthma from electronic health record (EHRs) systems. A scalable, efficient, and automated tool for ascertaining adult asthma status from EHRs remains nonexistent.

**Objective:**

We validated NLP-PAC enabling ascertainment and early identification of adult asthma status in their EHRs.

**Methods:**

We applied the validated NLP-PAC to EHRs of a convenient sample (adult cohorts who participated in our previous population-based studies) in which a reference standard (ie, asthma status defined by manual chart review) is available. The performance of NLP-PAC was assessed by determining criterion validity against manual chart review and construct validity before and after the new EHR (Epic) system was implemented in 2018.

**Results:**

The cohort consisted of 1,898 subjects, with 43% male and a median age at time of last follow-up of 65 years (interquartile range, 55-76). Manual chart review and NLP-PAC identified 97 (5.1%) and 98 (5.1%) subjects with asthma, respectively, with 89 subjects commonly identified by both methods. The sensitivity, specificity, positive predictive value, and negative predictive value of NLP-PAC were 92%, 99%, 91%, and 99%, respectively, before the new EHR system was implement, which remained similar after introducing the system (95%, 88%, 96%, and 85%, respectively). The risk factors for asthma identified either by NLP-PAC or manual chart review were similar.

**Conclusion:**

Automatic asthma ascertainment for adults based on EHR data is feasible with our NLP algorithm, offering immense scientific and clinical value for large-scale clinical research and population management for adult asthma care.

Asthma is a common chronic inflammatory respiratory disease affecting more than 20 million adults in the United States. Higher prevalences have been noted in adult women, persons who are Black, non-Hispanic multiple races, or Puerto Rican, as well as persons with low household income. On an annual basis, more than 8 million adults with asthma experience an asthma exacerbation, resulting in approximately 700,000 emergency department visits, 67,000 hospitalizations, and 3,300 deaths.^1^ Although effective, evidence-based management guidelines for asthma are readily available, inadequacies in asthma ascertainment could delay implementation of treatment strategies used to maintain symptom control and prevent exacerbations. In the absence of accurate asthma detection, the clinical burden of asthma will remain high and disparities in asthma morbidity will persist.^2^

As a result of these disparities, clinical and translational population-based asthma research is essential to advance asthma care. To be effective, asthma studies must overcome current issues including discordant asthma diagnostic criteria, inconsistent asthma ascertainment methodologies, and differing sampling frames. These core issues have rendered conflicting results in genome-wide association studies, clinical trials, and biomarker studies. One prime example of the downfall of current asthma research methodologies was noted by a prior study that reported that 60 different definitions for pediatric asthma have been used in 122 publications.^3^ If these issues are not adequately addressed, inconsistent study findings will continue and will in turn hinder the translation of such findings into clinical practice.

Presently, unstructured data, which are labor intensive to extract and record in a structured format, is estimated to account for >80% of health care data.^4^ Electronic health records (EHRs) offer a solution to the present difficulties with asthma research given their wide adoption in the United States, which enables large-scale data mining. Computational approaches to data analyses such as natural language processing (NLP) offer a vital opportunity to develop population-based asthma research tools. NLP can extract information from narrative text automatically, overcoming the limitations of structured data in EHRs and eliminating the effort and cost associated with manual chart review.^5^, ^6^, ^7^, ^8^ Automated algorithms for asthma identification are necessary to efficiently process large volumes of data and to apply standardized definitions for asthma identification.

Predetermined asthma criteria (PAC) for adults and children have been validated through asthma incidence and case–control studies,^9^, ^10^, ^11^, ^12^, ^13^, ^14^, ^15^ which provided the framework for our NLP algorithm, NLP-PAC. NLP-PAC allows for the expedient ascertainment of pediatric asthma status by extracting childhood asthma-relevant information from clinical notes and other unstructured text in EHRs.^16^, ^17^, ^18^, ^19^, ^20^, ^21^, ^22^, ^23^, ^24^, ^25^ The performance of NLP-PAC was comparable to manual review with a sensitivity, specificity, positive predictive value (PPV), and negative predictive value (NPV) of 97%, 95%, 90%, and 98%, respectively.^20^ Our prior groundwork illustrated the significant potential of NLP’s utilizing EHRs for population-based asthma research by applying consistent asthma criteria and ascertainment processes in a large-scale, efficient manner. Our previous success with identification of pediatric allergy paved the way for our current work to determine whether an NLP algorithm can be applied to EHRs to identify and ascertain asthma status in an adult population. Although the NLP-PAC algorithm was previously validated in pediatric populations, adapting it for adult asthma poses unique challenges as a result of differences in documentation patterns, broader differential diagnoses (eg, chronic obstructive pulmonary disease), and less standardized note structures, especially among older adults with multimorbidity. These differences necessitate rigorous validation of NLP-PAC in adult EHR data before widespread use. In doing so, we can better phenotype asthma from the EHR, identify asthma earlier in the disease course, and bridge the gaps that have been impeding population-level asthma research across the age spectrum.

## Methods

Our study protocol was approved by the institutional review boards at both Mayo Clinic and the Olmsted County Medical Center. This retrospective study utilized previous adult asthma study cohorts.^10^, ^11^, ^12^

### Study setting

All subjects resided in Olmsted County, Minnesota, and participated in the Rochester Epidemiology Project, which links all inpatient and outpatient clinical information from every episode of care to each patient and health care provider. Authorization for research use of existing medical record data is granted by >95% of all subjects who are registered with any health care providers in the community since 1966.^26^, ^27^, ^28^, ^29^, ^30^, ^31^

### Study design and subjects

This retrospective study leveraged data from 3 previously conducted population-based case–control studies that examined the association between asthma and adult inflammatory conditions, including zoster, myocardial infarction, and rheumatoid arthritis.^10^, ^11^, ^12^ These prior studies were selected because they: (1) applied the same asthma criteria (ie, PAC) via manual chart review, (2) utilized the EHR at Mayo Clinic, ensuring compatibility with our NLP-based approach, and (3) retained detailed documentation of asthma status, index dates, and known asthma risk factors, thus making them suitable for validating the NLP-PAC algorithm.

The current study included adults (≥18 years) from the original cohorts who had available EHR data from the year 2000 onward. We excluded (1) subjects who changed their research authorization (ie, from yes to no), (2) prevalent cases with index dates before 2000 (as determined by prior manual chart review), and (3) those with asthma-related diagnostic codes recorded outside the Mayo Clinic system (based on the Rochester Epidemiology Project Diagnosis index and established International Classification of Disease [ICD] version 9/10 code lists [eg, 493.xx, bronchospasm, bronchiolitis, wheezing, reactive airway disease codes]).^32^ To evaluate criterion validity, we applied the NLP-PAC algorithm to the EHRs of these 3 adult cohorts (2000 to the present) and compared the NLP-identified asthma cases with those previously identified by manual chart review (the reference standard). Notably, the original manual chart reviews included non-EHR records (before 2000), whereas NLP-PAC only accessed structured and unstructured data within the EHR system. As such, discrepancies due to data source differences were anticipated. In cases of disagreement, the reference standard was reevaluated on the basis of EHR data only, to ensure a fair assessment of NLP performance using a consistent data source. We also assessed construct validity by examining the association between NLP-ascertained asthma and known asthma risk factors captured in the original studies.

### PAC

The original PAC was developed and validated for retrospective studies among children and adults based on chart review (Table I).^9^ PAC was designed to apply to medical records to identify repeated respiratory symptoms of wheeze, cough, and/or dyspnea together with physiologic evidence of variable expiratory airflow limitation, which is the basis for asthma diagnosis and which is conceptually similar to the 2015 Canadian Thoracic and Canadian Pediatric Society asthma criteria consisting of (1) recurrent wheezing episodes or airflow obstruction, (2) reversibility via bronchodilator therapy, and (3) exclusion of alternative diagnoses.^33^ PAC is the only existing predetermined criteria for asthma that determine asthma status and the index date of incident asthma retrospectively on the basis of medical records for both adults and children. As defined by PAC, most cases of probable asthma (85%) became definite asthma over time,^9^^,^^34^ so we included both probable and definite asthma for our prior studies and current study. PAC has high reliability and has demonstrated excellent construct validity in identifying known risk factors for asthma and asthma-related adverse outcomes in numerous studies.^9^^,^^34^, ^35^, ^36^, ^37^, ^38^, ^39^, ^40^, ^41^, ^42^, ^43^ Index date was defined as the date when PAC was met for the first time. We did not exclude those who may have experienced asthma remission since onset because the scope of the study was to assess the precision of NLP compared to manual chart review for the same EHRs.

**Table I:** Predetermined asthma criteria

Patients were considered to have *definite* asthma if a physician had made a diagnosis of asthma and/or if each of the following 3 conditions were present, and they were considered to have *probable* asthma if only the first 2 conditions were present: 1. History of cough with wheezing, and/or dyspnea, *or* history of cough and/or dyspnea plus wheezing at examination, 2. Substantial variability in symptoms from time to time or periods of weeks or more when symptoms were absent, *and* 3. Two or more of the following: • Sleep disturbance by nocturnal cough and wheeze. • Nonsmoker (14 years or older). • Nasal polyps. • Blood eosinophilia of >300 eosinophils/μL. • Positive wheal and flare skin tests *or* elevated serum IgE. • History of hay fever or infantile eczema *or* cough, dyspnea, and wheezing regularly on exposure to an antigen. • Pulmonary function tests showing one FEV_1_ or FVC < 70% predicted and another with at least 20% improvement to FEV_1_ > 70% predicted *or* methacholine challenge test showing ≥20% decrease in FEV_1_. • Favorable clinical response to bronchodilator therapy. Patients were excluded from our previous study if any of these conditions were present: • Pulmonary function tests that showed FEV_1_ to be consistently <50% predicted or diminished diffusion capacity. • Tracheobronchial foreign body at or about incidence date. • Hypogammaglobulinemia (IgG < 2.0 mg/mL) or other immunodeficiency disorder. • Wheezing occurring only in response to anesthesia or medications. • Bullous emphysema or pulmonary fibrosis on chest radiograph. • PiZZ α_1_-antitrypsin. • Cystic fibrosis. • Other major chest disease such as juvenile kyphoscoliosis or bronchiectasis.

*FEV*_*1*_*,* Forced expiratory volume in 1 second; *FVC,* forced vital capacity.

### Development of NLP algorithm for PAC

**Fig 1:**
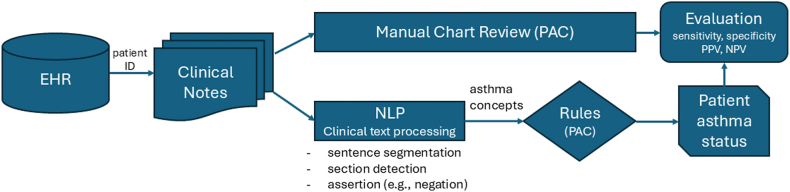
Algorithms of manual review and NLP-PAC system.

The development of the NLP algorithm for PAC was previously reported in detail,^19^^,^^20^^,^^22^ and the process for the NLP-PAC algorithm to ascertain asthma status is depicted in Fig 1. There are two basic components in NLP-PAC: (1) a clinical text processing component (extracts evidence text in EHRs to match in PAC delineated in Table I) and (2) a patient asthma classification component (classifies asthma status at a patient level using pattern-based rules, assertion status [eg, nonnegated (had wheezing vs denied wheezing), associated with patient (no family history)]), and section constraints (eg, diagnosis). Some primary concepts were combined into secondary concepts to meet the criteria (eg, “wheezing” and “coughing”). The algorithm was implemented using the open-source NLP pipeline MedTagger (https://github.com/OHNLP/MedTagger) developed by Mayo Clinic.^44^

### Concordance in asthma ascertainment between NLP algorithms and manual review (criterion validity)

We applied NLP-PAC to the EHRs of eligible subjects to ascertain asthma status. Each subject had multiple EHR notes documented by multiple health care providers (eg, nurse note, physician note) during the study period. In order to compare the asthma status defined by NLP-PAC with the reference standard of manual chart review, we utilized existing asthma status and its index date by PAC, which was obtained from the previous 3 population-based case–control study cohorts as described above.^10^, ^11^, ^12^ The data abstractors were unaware of the asthma status determined by the NLP algorithm. Any initial discrepancies were adjudicated by two independent reviewers (H.S., a pulmonology specialist; and M.P., an allergy/immunology specialist) and corrected if the initial annotation by human was not correct. Sensitivity, specificity, PPV, and NPV were reported for criterion validity.

### Asthma risk-factor variables (construct validity)

*Construct validity* refers to the degree to which a test or other measure assesses the underlying theoretical construct it is supposed to measure—that is, the test is measuring what it is purported to measure.^20^^,^^45^ The reported risk factors for asthma, including a history of allergic rhinitis, history of atopic dermatitis, and smoking status, were collected during the original population-based case–control studies^11^^,^^12^^,^^46^ and utilized for this study to assess construct validity (ie, the association between asthma status by NLP and abstractor for comparison purposes).

### Adaptation of NLP-PAC for the new Epic EHR system

Adapting existing NLP algorithms to different health care settings is reportedly both challenging and successful.^23^^,^^47^ Even within a single health care setting, the change of EHRs requires adjustments in NLP or other artificial intelligence algorithms as part of postimplementation maintenance. Before 2018, we developed, validated, and implemented NLP-PAC from Mayo Clinic’s EHRs.^20^ Since Mayo Clinic transitioned from a GE-based EHR system to the Epic EHR system in 2018, we further validated NLP-PAC against post-Epic EHRs to ensure it functions with the new EHR system. First, we carefully assessed the adaptation process of NLP-PAC^20^ and revalidated the performance in the new Epic EHR system with the reference standard of human chart review in 303 randomly selected children from the Mayo Clinic birth cohort between June 2018 to December 2019. One hundred fifty-two EHRs were used for the adjustment and refinement of the NLP-PAC algorithm, and 151 were used for the final validation. Then we randomly sampled 100 adults whose EHRs existed in both the pre- and post-Epic eras with 80 subjects with ICD-10 codes for asthma (J45.xx) and 20 subjects without, and we then validated the performance of NLP-PAC in the new Epic EHR system (June 2018 to December 2019).

### Statistical analysis

The performance of NLP-PAC was assessed for both criterion and construct validity. For criterion validity, we calculated unweighted Cohen kappa index, agreement rate, sensitivity, specificity, PPV, and NPV for concordance in asthma status between NLP-PAC and updated manual chart review through error analysis as the reference standard. Because of the complex and haphazard sampling design (ie, combination of 3 prior case–control studies on adults), these are estimates of population parameters but give a sense of how well NLP-PAC reproduces manual chart review in a common research setting. Similarly, because the standard assumptions for statistical inference are violated by the sampling design, we do not provide confidence intervals. The same analysis was conducted for the adaptation process for the new Epic EHR system. Using logistic regression models, construct validity was tested by assessing the association of NLP-PAC results with the known common risk factors for asthma (eg, age, sex, race/ethnicity, history of allergic rhinitis and eczema, and smoking status) because NLP-PAC is expected to be correlated with the known risk factors for asthma if it captures the underlying construct (ie, asthma). The construct validity of NLP-PAC was compared to that of an updated manual chart review of the EHR. The associations were summarized by calculating odds ratios and corresponding 95% confidence intervals. Statistical analyses were performed by JMP v10 software (SAS Institute, Cary, NC).

## Results

### Study subjects

Of the 2,595 subjects from the original studies who granted research authorization, 697 were excluded for the following reasons: 360 had an asthma index date before 2000, 95 lacked EHRs since 2000, and 242 had asthma-related diagnostic codes recorded outside Mayo Clinic. Among the 1,898 eligible study subjects, 820 (43%) were male, 1,777 (94%) were White, and the median (range) age at the last follow-up date was 65 (20-99) years (interquartile range, 55-76) (Table II).

**Table II:** Characteristics of 1,898 study subjects

Characteristic	Value
Age (years) at last follow-up date, median (IQR)	65 (55, 76)
Male sex	820 (43)
Race	
White	1777 (94)
Unknown	60 (3)
Asian	45 (2)
Black	16 (1)
Allergic rhinitis	213 (11)
Eczema	374 (20)
Smoking status	
Never	983 (52)
Current	236 (12)
Former	661 (35)
Unknown	18 (1)

Data are presented as nos. (%) unless otherwise indicated. *IQR,* Interquartile range.

### Criterion validity

#### Concordance between NLP-PAC and chart review

Among the 1,898 subjects, the NLP-PAC algorithm identified 98 subjects as meeting asthma criteria and manual chart review identified 97, with 89 cases overlapping. Agreement metrics between NLP-PAC and chart review showed excellent concordance, with a kappa value of 0.91 and overall agreement of 0.99 (Table III). Using chart review as the reference standard, NLP-PAC had a sensitivity of 92%, specificity of 99%, PPV of 91%, and NPV of 99%.

**Table III:** Confusion matrix of showing agreement between NLP-PAC and reference standard manual chart review

Manual chart review	NLP-PAC		Row percentage
	Asthma	No asthma	
Asthma	89	8	Sensitivity = 92
No asthma	9	1792	Specificity = 99
Column percentage	PPV = 91	NPV = 99	Accuracy = 99

#### NLP-PAC validation across EHR systems

Before applying NLP-PAC to adults in the new Epic EHR system, we first retested the adapted algorithm in a pediatric cohort (n = 151) with a median age of 9.2 years (interquartile range, 3.1-12.8). NLP-PAC identified 80 subjects with asthma (vs 81 by human annotators), resulting in sensitivity of 97%, specificity of 98%, PPV of 93%, and NPV of 99%, similar to the performance on the previous GE-based EHR.^20^ Subsequently, NLP-PAC was validated in adults using the Epic EHR system. In this setting, the algorithm obtained sensitivity of 95%, specificity of 88%, PPV of 96%, and NPV of 85%, consistent with results from the earlier system. Subsequently, NLP-PAC was validated in adults using the result for the new EHR system. In this setting, the algorithm obtained sensitivity of 95%, specificity of 88%, PPV of 96%, and NPV of 85%, consistent with results from the earlier system (Table III).

### Construct validity

For association with known risk factors, construct validity was assessed by examining the relationship between asthma status (as determined by NLP-PAC and chart review) and known risk factors (Table IV). Asthma status by both NLP and manual chart review were significantly more likely to have a history of allergic rhinitis and eczema (*P* < .05 for both). No significant associations were found with sex, race, and smoking status. These patterns were consistent across both NLP-PAC and manual chart review, supporting the construct validity of the algorithm.

**Table IV:** Associations of asthma status determined by NLP-PAC and abstractor with known risk factors for asthma (N = 1,898)

Characteristic	By NLP-PAC				By manual chart review			
	No asthma (n = 1,800)	Asthma (n = 98)	OR (95% CI)	*P* value	No asthma (n = 1,801)	Asthma (n = 97)	OR (95% CI)	*P* value
Age (years) at last follow-up, median (IQR)	65 (55, 76)	65 (53, 77)	1.0 (0.9, 1.0)	.97	65 (55, 76)	65 (54, 77)	1.0 (0.9, 1.0)	.82
Male sex	783 (44)	37 (38)	0.8 (0.5, 1.5)	.26	784 (44)	36 (37)	0.8 (0.5, 1.2)	.21
White	1683 (94)	94 (96)	1.6 (0.6, 4.5)	.33	1684 (94)	93 (96)	1.6 (0.6, 4.5)	.35
Allergic rhinitis	195 (11)	18 (18)	1.9 (1.1, 3.2)	.02	194 (11)	19 (20)	2.0 (1.2, 3.4)	.007
Eczema	337 (19)	37 (38)	2.6 (1.7, 4.0)	<.001	338 (19)	36 (37)	2.6 (1.7, 3.9)	<.001
Smoking status								
Never	937 (52)	46 (47)	Ref	Ref	936 (52)	47 (48)	Ref	Ref
Current	221 (12)	15 (15)	1.4 (0.8, 2.6)	.29	221 (12)	15 (15)	1.4 (0.7, 2.4)	.32
Former	624 (35)	37 (38)	1.2 (0.8, 1.9)	.40	626 (35)	35 (36)	1.1 (0.7, 1.7)	.64
Unknown	18 (1)	0	NA	NA	18 (1)	0	NA	NA

Data are presented as nos. (%) unless otherwise indicated. For example, subjects identified with asthma by PAC compared to those without asthma had higher odds of history of allergic rhinitis and eczema (*P* < .05 for both). These patterns were consistent across both NLP-PAC and asthma status by manual chart review, supporting algorithm’s construct validity. *CI,* Confidence interval; *IQR,* interquartile range; *NA,* not applicable; *OR,* odds ratio.

## Discussion

Our study is the first to have developed an NLP algorithm that accurately and efficiently determines adult asthma status while also addressing the noted challenges of asthma diagnosis, characterization, and ascertainment. We demonstrated that ascertainment of adult-onset asthma by our NLP algorithm utilizing EHR data has excellent concordance with manual chart abstraction (criterion validity) and demonstrates significant association with known risk factors for asthma (construct validity). These findings provide a framework to identify adult patients with asthma with large-scale throughput, efficiency, and reproducibility, thereby enabling clinicians and researchers to leverage previously untapped free-text information in EHRs to advance asthma care.

No tools are currently available for population-level research for adult asthma ascertainment that effectively mitigate issues involving inconsistent asthma diagnostic criteria and heterogenous asthma ascertainment methodologies. To address the current shortcomings in clinical and translational population-based asthma research, our NLP algorithm needed to excel in several aspects. Criterion validity indexes for our NLP algorithm demonstrated strong agreement with a kappa index and agreement for asthma status between NLP-PAC and chart review by annotators of 0.91 and 0.99, respectively. Asthma status determined by NLP-PAC performed similarly to that of manual chart review for these known asthma risk factors (construct validity). The lack of significance for certain factors like smoking status may be due to limited power or cohort characteristics and warrants further investigation in larger or more diverse populations. The use of PAC in our NLP algorithm lends considerable strength to our excellent criterion and construct validity, as PAC has demonstrated high reliability and construct validity across multiple studies over years of extensive epidemiologic work for asthma.^9^^,^^34^, ^35^, ^36^, ^37^, ^38^, ^39^, ^40^, ^41^, ^42^, ^43^

Prior studies utilizing NLP for identifying adult asthma patients in the EHR are limited. A recent systematic review of NLP-based research in asthma identified 13 publications in which NLP was primarily used for determining asthma status.^5^ Of these 13 publications, only one involved adults,^48^^,^^49^ while the remaining studies primarily focused on pediatric cohorts. In the lone adult asthma NLP-based study, Himes et al^48^ extracted data from an asthma registry consisting of 12,792 adult asthma patients. This registry contained structured data from patient records such as billing codes, as well as additional concepts obtained by using NLP on unstructured textual notes of medical records. ICD-9 codes were used for asthma ascertainment, and the extracted data were used to predict which patients experienced frequent asthma exacerbations. A multivariable logistic regression model for asthma exacerbations was created with an area under the receiver operating characteristic curve, or AUROC, score of 0.67, which was not acceptable as a clinical classifier. The inadequate performance of this model likely stems from the lack of criterion and construct validity that was a strength of our NLP algorithm.

Another recent systematic review evaluated 67 studies that used automated detection of obstructive lung diseases through imaging, genetics, auditory signals, airflow data, or the EHR.^50^ Of the 67 studies reviewed, only 3 studies used EHR-based data to evaluate adult asthma patients, and none of these 3 studies used NLP methodology. Either machine learning or deep learning was used in these 3 studies, with sensitivities ranging from 75% to 91% for identification of asthma or asthma exacerbation. The performance of these computational learning models is subject to the same challenges facing population-based asthma research, including inconsistent asthma diagnostic criteria, methodologic heterogeneity of asthma ascertainment, and differing sampling frames. Our NLP-PAC was able to address these challenges.

A third systematic review of artificial intelligence techniques in asthma reviewed 98 publications that were categorized as either asthma screening and diagnosis, patient classification, asthma management, or asthma treatment.^51^ Of these 98 studies, only one utilized NLP to process EHR-based data. However, this single study focused on asthma status identification in children and was conducted by our research group.^18^

In summary, our study is unique in the use of NLP for EHR-based data focusing on asthma ascertainment in adults. Identifying such a cohort may not be feasible with structured data alone (eg, diagnosis and billing codes). Furthermore, we show that NLP has the capability to capture temporal components of clinical notes and can thereby determine important events such as dates of asthma onset, remission, and relapse.^25^ Given the volume of available EHR-based data, NLP and other artificial intelligence approaches for data mining and knowledge discovery should be developed and implemented in education, clinical practice, and research. In particular, large language models have received considerable attention for their ability to recognize, interpret, and generate text with minimal adjustment of the training parameters. With continued refinement, large language models may facilitate language analyses at much greater scales than previously possible.^52^

Our study has several strengths. First, we developed and advanced our NLP algorithm for incorporating unstructured data. Second, our NLP algorithm can determine temporal relationships of events of interest, such as index date for asthma. Third, we used the Rochester Epidemiology Project dataset to conduct retrospective, population-based studies in which all outpatient and inpatient asthma-related events can be captured with longitudinal follow-up throughout the lifetime. Last, we used a PAC that is well validated for asthma ascertainment. Accordingly, our study highlights the use of NLP algorithms for adult asthma ascertainment for population-level EHR-based research, serving to mitigate issues involving various inconsistent asthma diagnostic criteria and heterogeneous asthma ascertainment procedures.

Our study does have a number of limitations that limit the generalizability of the findings to other health care settings or populations. The study cohort had limited diversity and was restricted to those patients living and receiving health care in Olmsted County, Minnesota. Thereby, variability in asthma prevalence or risk factors across different racial groups is not accounted for in our analysis. In addition, the cohort was based on a convenience sample rather than a random or population-based sample, and our study utilized retrospective data without prospective or cross-sectional data for validation. Finally, potential misclassification of asthma status may occur by using PAC. When considering these limitations, we acknowledge that our NLP algorithm represents developmental work and may have portability issues at other sites. Our construct validity assessment was limited to a few binary risk factors as a result of constraints in the original datasets, and future studies with more comprehensive data are needed for robust multivariable validation. A small sample size for validating for adaptation of EHRs in adults was also a limitation.

Our study results will need to be replicated and validated in different study settings to determine the accuracy of our NLP algorithm for adult patients with asthma. As a model, our prior work using NLP algorithms for pediatric asthma has been validated in multiple different institutions’ EHRs and is now being deployed as a clinical decision support tool for childhood asthma management.^53^ Regarding potential asthma misclassification, PAC has been used extensively in epidemiologic work for asthma and has been found to have high reliability as well as excellent construct validity. Furthermore, we have performed several pediatric studies^18^, ^19^, ^20^, ^21^, ^22^, ^23^^,^^25^^,^^54^^,^^55^ that utilized PAC to identify pediatric asthma from the EHR via an NLP algorithm. In validating NLP-PAC in children, we reported high criterion validity, including 97%, 95%, 90%, and 98% sensitivity, specificity, PPV, and NPV, respectively,^20^ which were similar to the current study in adults. In addition, our prior work on pediatric asthma demonstrated that PAC clearly differentiates those with and without asthma both clinically and immunologically.^56^

In conclusion, our NLP algorithm constructed for automated chart review for adult-onset asthma ascertainment in the EHR is as accurate and more efficient than manual chart review. Our NLP-PAC algorithm is an innovative and useful tool enabling large-scale clinical studies for research and population management for asthma care in adults. Although our results need to be replicated with additional studies with larger sample sizes using different EHR systems, our study results suggest the potential of expanding the use of NLP for adult asthma research and practice in the era of EHRs and big data.

## Disclosure statement

Supported by the 10.13039/100000002National Institutes of Health (grants R01 HL126667, R21 AI142702, and R21 AG65639).

Disclosure of potential conflict of interest: The authors declare that they have no relevant conflicts of interest.
