# Supplementary material for: Application of a natural language processing algorithm to early asthma ascertainment for adults in the era of electronic health records
Source: J Allergy Clin Immunol Glob. 2025 Nov 26;5(2):100618. doi: 10.1016/j.jacig.2025.100618 (PMC12769801; doi:10.1016/j.jacig.2025.100618)
Supplement: Supplementary Materials [file mmc1.docx]

**Supplementary document**

*Adaptation of Natural Language Processing Algorithms for Childhood Asthma in Response to Change of EHR System:* We first assessed the adaptation process of two existing NLP algorithms for children (NLP-Predetermined Asthma Criteria [PAC] and NLP-Asthma Predictive Index [API])^17,19^ and revalidated the performance in the new EHR System (Epic). The study was designed as a retrospective cohort study using a subset of the Mayo Clinic birth cohort. We adapted NLP-PAC and NLP-API on a weighted random sample of 303 children’s EHRs (Epic) between June 2018 to December 2019. Since the study period was relatively short (1.5 years), we attempted to identify more children with asthma using ICD-10 codes: (1) 204 children with asthma by ICD-10 (J45) since birth, (2) 49 children with asthma-related diagnosis (e.g., wheezing (R06.2), cough (R05)), bronchiolitis (J21), bronchitis (J20), shortness of breath (R06.0X), flu (J09-J11), pneumonia (J12-J18)), but without ICD-10 code of J45, and lastly, (3) 50 children without any ICD-10 codes for asthma or asthma-related diagnosis. This process ensures inclusion of reasonable numbers of EHRs relevant to asthma that are required to refine and adapt NLP algorithms. Out of 303 children, 152 EHRs were used for adjustment and refinement of NLP algorithms, and 151 for the final validation.

During the Ad*justment Phase,* the NLP algorithms were adjusted to address Epic-specific variations occurring in the clinical notes and the lab data standard (e.g., section tag in clinical notes, laboratory data format) to ensure technical operability in the new EHR system. During the *Refinement Phase*, the NLP algorithms were refined to fix incorrectly identified cases in the Epic EHR. Most incorrect cases were due to assertion status (e.g., negation, hypothetical). Also, newly identified description patterns of asthma-related concepts were added to NLP algorithms. Lastly, *validation phase*, the performance of the adapted NLP-PAC and NLP-API was assessed against manual chart review (e.g., sensitivity, specificity, positive predictive value (PPV), and negative predictive value (NPV)).

The median age of the validation cohort was 9.2 years (interquartile range: 3.1-12.8). NLP-PAC and NLP-API, after the final adaptation, identified 80 and 16 subjects with asthma, respectively (vs. 81 and 17 by human annotators). Sensitivity, specificity, PPV, and NPV in asthma ascertainment were 97%, 98%, 93%, and 99%, respectively for NLP-PAC; and 94%, 100%, 100%, and 98%, respectively for NLP-API, which are similar to performance of the original algorithms developed on the previous EHR System (GE-based EHR).^17,19^ The performance of the NLP algorithms after the initial adjustment phase (i.e., out-of-box algorithms except adjusting Epic EHR variations to make it technically operable) was not as good as the original NLP algorithms.
